# Memantine effects on liver and adrenal gland of rats exposed to cold stress

**DOI:** 10.1186/1755-7682-4-5

**Published:** 2011-01-22

**Authors:** Marcelo Ferreira, Vitor E Valenti, Jose R Cisternas, Celso Ferreira, Adriano Meneghini, Celso Ferreira Filho, João R Breda, João A Correa, Carlos Bandeira de Mello Monteiro, Hugo Macedo Junior, Neif Murad, Luiz Carlos de Abreu

**Affiliations:** 1 Laboratório de Escrita Científica, Departamento de Morfologia e Fisiologia, Faculdade de Medicina do ABC, Santo André, SP, Brasil; 2 Departamento de Clínica Médica, Disciplina de Cardiologia, Faculdade de Medicina do ABC, Santo André, SP, Brasil; 3 Disciplina de Cirurgia Vascular, Faculdade de Medicina do ABC, Santo André, SP, Brasil; 4 Division of Endocrinology, Metabolism and Diabetes, University of Utah, School of Medicine, Salt Lake City, Utah, USA; 5 Escola de Artes, Ciência e Humanidades da Universidade de São Paulo (USP), São Paulo, SP, Brasil

## Abstract

**Background:**

Memantine attenuates heart stress due cold stress, however, no study focused its effects on liver and adrenal gland. We evaluated its effects on lipid depletion in adrenal gland and glycogen depletion in liver of rats exposed to cold stress.

**Methods:**

Male rats divided into 4 groups: 1)Control (CON); 2)Memantine (MEM); 3)Induced cold stress (IH) and; 4)Induced cold stress memantine (IHF). Memantine were administrated by gavage (20 mg/kg/day) during eight days. Cold stress were performed during 4 hours once at - 8°C. Lipid and glycogen depletion were presented as its intensity levels.

**Results:**

Rats exposed to cold stress presented the highest glycogen (p < 0.001) and lipid depletion (p < 0.001) in liver and adrenal gland, respectively. We noted that memantine significantly reduced lipid depletion in adrenal gland and glycogen depletion in liver.

**Conclusion:**

Memantine prevented glycogen depletion in liver and lipid depletion in adrenal gland of rats under a cold stress condition.

## Background

More than 60 years ago, Selye [1] recognized that the physiological system was activated by stress, and that protecting and restoring the body could also damage the system [2-5]. Induced cold state by low temperature exposure is considered as an important stressing physical agent [6]. Previous studies reported that acute exposure to cold stress besides impair heart tissue [7-9] cause lipid depletion in adrenal gland and glycogen depletion in liver [10], suggesting that the sympathetic activity and oxidative stress are the source of such fact.

It is well established that glutamate excitotoxicity triggers neurodegeneration in acute brain injury conditions such as stroke, status epilepticus and head trauma. Drugs that block N-methyl-d-aspartate (NMDA) glutamate receptors are known to be neuroprotective in animal models for studying these acute brain injury conditions [11,12]. Memantine is a non-competitive antagonist at NMDA receptors and it is currently used to treat subjects with moderate to severe Alzheimer's disease to improve cognitive symptoms [13].

Although it was indicated that glutamate receptors antagonists reduces oxidative stress mechanism in neurologic diseases [14], no study focused its effects on liver and adrenal gland in an acute cold stress situation. Furthermore, considering that increased sympathetic activity is also involved in physiological stress responses [1] we hypothesized that pretreatment with a NMDA-antagonist would reduce the glycogen and lipid depletion in liver and adrenal gland, respectively. Therefore, this investigation was undertaken to evaluate the effects of memantine treatment on lipid depletion in adrenal gland and glycogen depletion in liver.

## Method

### Animals

Experiments were performed on 40 adult male rats (Rattus *novergicus albinus*, Rodentia Mammalia), EPM-Wistar, weighing 230-340 grams. Rats were obtained from the Central Biotery of our University. Temperature was monitored as 22°C, air humidity nearly 60% and the clear-dark cycle was controlled and established as twelve hours each one. Animals had free access to food and water. After an adaptation period of nearly one week animals were randomly selected and separated into four groups: Control Group (CON, n = 10): rats treated by the administration of gavages containing 1 mL of water at 10:00 a.m. during 8 consecutive days; Memantine Group (MEM, n = 10): rats treated by the administration of gavages (1 mL) containing 20 mg/kg memantine at 10:00 a.m. during 8 consecutive days; Stress Group (S, n = 10): rats treated by the administration of gavages containing 1 mL of water at 10:00 a.m. and exposed to -8°C during four hours on the last day (8^th^) and; Memantine + Stress Group (MEM+S, n = 10): rats treated by the administration of gavages (1 mL) containing 20 mg/kg memantine at 10:00 a.m. during 8 consecutive days and exposed to -8°C during four hours on the last day (8^th^). All procedures were performed in accordance with ethical guidelines of the National Institutes of Health Guide for the Care and Use of Laboratory Animals and were approved by the Ethical Committee in research of our University (number 1029/06).

### Cold Stress Procedure

Rats were exposed to cold stress by maintaining them at -8°C during 4 hours in a refrigerated compartment in wire mesh cages. Cold stress was performed only once. Rat's temperature was controlled at approximately 37°C and the behavior of the animals was observed during all the procedures [5,6,15,16].

### Histological Procedure

After adequate level of ether anesthesia, we verified tail tonus and response to external stimuli before and during surgical procedure through evaluation of vibrissa movements; all animals were submitted to a laparotomy. Two pieces of the left liver lobe and right adrenal gland were removed for light microscopy investigation. Regarding glycogen, fragments were dipped in Gender liquid at 4°C during 24 hours and were cut into small pieces of 1 mm^3 ^and post-fixed in a 1% OsO_4 _solution for 2 hours, dehydrated and embedded in araldite. Silver or gray thin sections (60-90 nm) were selected on a Porter-Blum MT-B ultramicrotome. The ultra-slices were mounted on copper silver grids with 200 patches and stained with uranyl acetate and lead citrate. In relation to lipid, fragments were fixed in formaldehyde 10% at 4°C. Approximately 24 hours later they were cut in cryostat. In order to indentify glycogen under light microscopy the cuts were submitted to Periodic acid-Schiff (PAS) after rehydration. PAS is a staining method used to detect glycogen in tissues. The reaction of periodic acid selectively oxidizes the glucose residues, creates aldehydes that react with the Schiff reagent and creates a purple-magenta color. A suitable basic stain is often used as a counterstain [17]. Lipids were stained by the method of Sudan IV in order to be evaluated by light microscopy. Sudan IV (C24H20N4O) is a lysochrome (fat-soluble dye) diazo dye used for the staining of lipids, triglycerides and lipoproteins on frozen paraffin sections. It has the appearance of reddish brown crystals with melting point 199°C and maximum absorption at 520(357) nm [18]. Glycogen depletion in hepatocytes and lipid depletion in adrenal gland cells were evaluated by three different investigators. We presented data concerning lipid or glycogen depletion through intensity levels (+ = small intensity; ++++ = high intensity/-= 0; + = 1; ++ = 2; +++ = 3; ++++ = 4). Samples were examined by three independent investigators with the same and standardized criteria. Histological slides were reviewed by a pathologist. Previous publications validated this procedure [5,6,19].

### Statistical Analysis

In order to evaluate the data associated to lipid depletion in adrenal gland cells and glycogen depletion in hepatocytes, comparison among independent groups, Kruskall-Wallis and Tukey post-hoc tests were applied. Concordance of measurements performed by the three investigators was evaluated and analyzed by *Bartko's intra-class correlation coefficient according to Fleiss guidelines [20], which was validated in previous studies [7,9,15,16] (p < 0.05, level of significance).

*Bartko's test formula: R = N(PMS - EMS)/N(PMS) (K - 1) (RMS) (N - 1)(K - 1)EMS

R = Bartkos correlation index; PMS = Patients Mean Square; RMS = Reasearcher Mean Square; EMS = Error Mean Square; N = Number of events; K = Number of investigators

## Results

After the cold stress exposure procedure we observed no difference between the memantine group and the not treated group regarding their behavior. Body weights were not statistically different among the groups (Stress group-236.8 ± 8.7; Control group-234.8 ± 14.94; Memantine group-241.2 ± 5.67; Memantine stress group-246.9 ± 12.57; p > 0.05).

**Figure 1:**
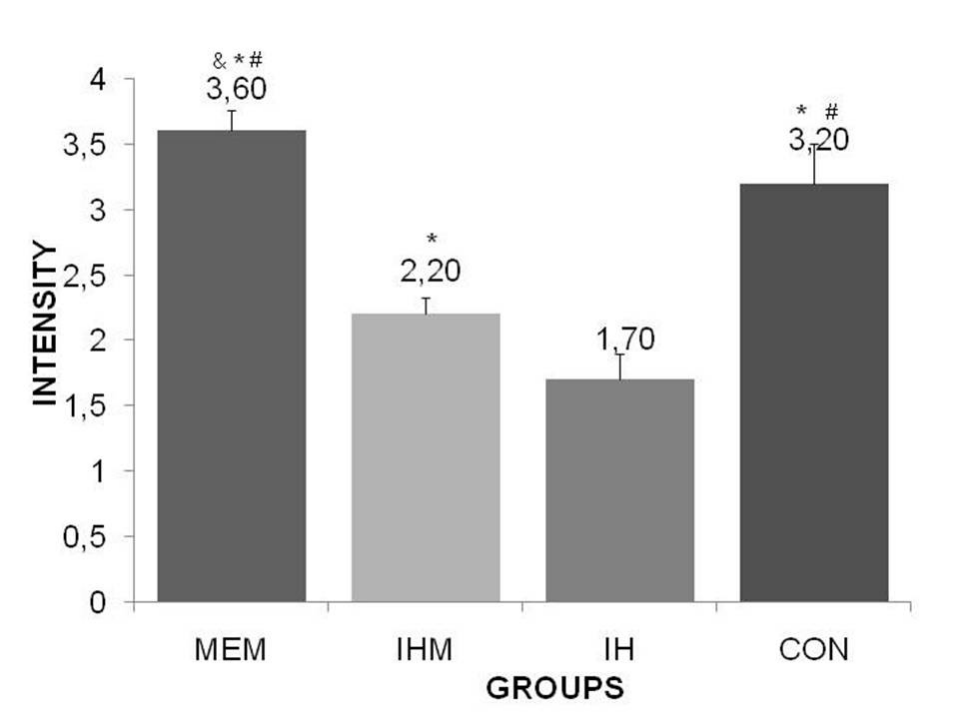
**Intensity of positivism regarding of glycogen depletion in hepatocytes (blushed with PAS method) of Control (CON), Induced Hypothermic (IH), Memantine pretreatment (MEM) and Induced Hypothermic Memantine pretreatment (IHM) groups**. + = small intensity; ++++ = high intensity/-= 0; + = 1; ++ = 2; +++ = 3; ++++ = 4. *p < 0.001; different from IH group. ^&^p < 0.01; different from CON group. ^#^p < 0.001; different from IHM group.

**Figure 2:**
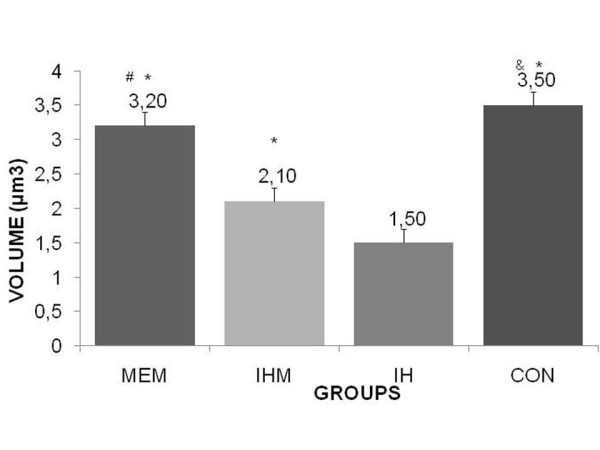
**Intensity of positivism regarding lipid depletion in adrenal gland cortical cells (blushed with Sudan IV) of Control (CON), Induced Hypothermic (IH), Memantine pretreatment (MEM) and Induced Hypothermic Memantine pretreatment (IHM) groups**. + = small intensity; ++++ = high intensity/-= 0; + = 1; ++ = 2; +++ = 3; ++++ = 4. *p < 0.001; different from IH group. ^&^p < 0.01; different from MEM group. ^#^p < 0.001; different from IHM group.

The induced hypothermic group presented the most increased rate of glycogen depletion in hepatocytes (Figure 1) (Table 1) (p < 0.05) and the most increased rate of lipid depletion in adrenal gland cortical cells (Figure 2) (Table 2) (p < 0.05), it supports that the exposure to -8°C during four hours was effective as a stressing agent. On the other hand, the induced hypothermic memantine group presented slight preserved lipid depletion in adrenal gland (Table 2) and glycogen depletion in liver (Table 1). Our findings indicate that memantine significantly reduces lipid depletion in adrenal gland and glycogen depletion in liver. Data variance analysis by *Bartko's *correlation index ranging between 0.43-0.93 in all experimental groups depicts the methodology validation. Moreover, variation analysis among group's means differences was statistically significant (p < 0.05).

**Table 1:** Glycogen depletion intensity in hepatocytes blushed with PAS method.

*Animal*	*Stress*	*Control*	*Memantine*	*Memantine + Stress*
***1***	**++**	**+++**	**++++**	**++**
***2***	**+**	**+++**	**++++**	**++**
***3***	**++**	**++++**	**+++**	**+++**
***4***	**+**	**++++**	**+++**	**+++**
***5***	**++**	**+++**	**+++**	**++**
***6***	**++**	**+++**	**++++**	**+++**
***7***	**+**	**++++**	**++++**	**++**
***8***	**++**	**++++**	**++++**	**+++**
***9***	**+**	**+++**	**+++**	**+++**
***10***	**+**	**++**	**++++**	**++**

+ = Small intensity (higher glycogen depletion); ++++ = High intensity (smaller glycogen depletion). + = Intensity of positivism.

**Table 2:** Lipid depletion intensity in adrenal gland cortical cells blushed with Sudan IV.

*Animal*	*Stress*	*Control*	*Memantine*	*Memantine + Stress*
***1***	**+**	**+++**	**+++**	**+++**
***2***	**++**	**++++**	**++++**	**+++**
***3***	**+**	**+++**	**++++**	**+++**
***4***	**+**	**++++**	**+++**	**++**
***5***	**++**	**+++**	**++++**	**+++**
***6***	**+**	**++++**	**+++**	**+++**
***7***	**+**	**+++**	**+++**	**+++**
***8***	**+**	**++++**	**+++**	**+++**
***9***	**++**	**+++**	**+++**	**+++**
***10***	**+**	**+++**	**+++**	**++**

+ = Small intensity (higher lipid depletion); ++++ = High intensity (smaller lipid depletion). + = Intensity of positivism.

## Discussion

We endeavored to investigate the effects of memantine pretreatment on lipid depletion and glycogen depletion in adrenal gland and liver cells, respectively, in rats acutely exposed to cold stress. It was observed that cold stress exposure increased lipid depletion in adrenal gland and glycogen depletion in hepatocytes, which supports previous studies [5,6]. We noted that this NMDA antagonist, currently used to treat subjects with moderate to severe Alzheimer's disease [4,5], significantly attenuated glycogen and lipid depletion in liver and adrenal gland, respectively, caused by cold stress in health Wistar rats.

The behavioral analysis allowed us to observe stress manifestation in rats after they were exposed to -8°C. Their behavior was similar to the initial reaction proposed by Selye et al [1]. Murad et al [21] noticed similar reactions. In addition, our histological analysis of liver tissue showed lower concentration of glycogen on hepatocytes of rats exposed to cold stress. This is explained by the fact that oxidative phosphorylation promotes glycogenolysis acceleration in response to catecholamine release caused by a stress condition, which lead to a lower concentration of glycogen on the cortical area of liver cells [22], such as we found in our study. The histological analysis also demonstrated that the stress group presented higher depletion of lipids on the cortical area of adrenal gland cells. This fact is explained by catecholamine release, which stimulates lipolysis in adrenal gland cells [22].

The effect of memantine on glycogen depletion observed in our findings could be explained by its antioxidants properties. A recent study [14] cited the importance of the various treatments based on NMDA-receptor antagonists including those targeted towards oxidative stress and excitotoxicity regarding Ca^2+ ^homeostasis. Furthermore, a previous investigation noticed that various NMDA-receptor antagonists (memantine was one of them) concentration-dependently diminished all oxidase model reactions in rat liver [23]. This research supports our data that memantine possibly reduces the effects of cold stress on liver cells through its antioxidant property.

Other explanation for our results is the fact that in animals glutamatergic NMDA signaling has been shown not only to be essential for synaptic plasticity underlying memory formation, but also for the regulation of neuroendocrine secretion [24]. Glutamatergic signaling is involved in the control of the hypothalamic-pituitary-adrenocortical (HPA) axis such that intraventricular administration of glutamate increases activity of the HPA system [25] and contributes to stress induced hormone release [26,27]. Our study supports those data, although we did not measure plasmatic catecholamines, the histological analysis provides evidence that glutamate blocker treatment with memantine reduced the physiological stress responses to cold exposure in adrenal gland and hepatocytes of rats due attenuated lipid and glycogen depletion, respectively.

Bähr et al [27] commented the need for antioxidants to protect the function of the adrenal cortex against reactive oxygen species generated by lipid peroxydation in the adrenal cortex, however, to our knowledge, no study evaluating direct effects of memantine on adrenal gland was observed. A previous research reported an inhibitory effect of antidepressant drugs on the corticosterone-induced promoter gene activity and suggested that the decrease in the glucocorticoid receptor-mediated gene transcription produced by antidepressants may be a mechanism by which these drugs block some effects induced by stress or corticosterone administration [28]. This investigation supports our data, since the reduction in the glucocorticoid receptor-mediated gene transcription induced by glutamate antagonist prevents the effects of stress [28].

Our study provides relevant information to the literature, since previous investigations have already indicated the importance of liver [29-31] and adrenal gland [32] for survivor. Other mechanism that could explain memantine effects on glycogen depletion in liver and lipid depletion in adrenal gland is its role on autonomic function. The possibly reduction of sympathetic activity caused by this drug decrease catecholamine release, which lead to a higher concentration of glycogen on the cortical area of liver and also a higher concentration of lipids on the cortical area of adrenal gland cells [22]. Moreover, Drever et al [33] suggested actions of memantine beyond NMDA receptor antagonism, including stimulating effects on cholinergic signalling via muscarinic receptors, therefore, reducing catecholamine levels.

In conclusion, our findings showed that memantine significantly reduced glycogen depletion in liver and lipid depletion in adrenal gland under an acute cold stress condition. Thus, we suggest that memantine prevents liver and adrenal gland from an acute cold stress exposure in rats.

## Competing interests

The authors declare that they have no competing interests.

## Authors' contributions

MF, VEV, JRC, CF, AM, CFF, JRB, JAC, CBMM, HMJ, NM and LCA designed research, analyzed data, and wrote the paper. All authors read and approved the final manuscript.
