# Correction: memantine effects on liver and adrenal gland of rats exposed to cold stress

**DOI:** 10.1186/1755-7682-4-7

**Published:** 2011-02-21

**Authors:** Marcelo Ferreira, Vitor E Valenti, Jose R Cisternas, Celso Ferreira, Adriano Meneghini, Celso Ferreira Filho, João R Breda, João A Correa, Carlos Bandeira de Mello Monteiro, Hugo Macedo Junior, Neif Murad, Weslei de Souza, Luiz Carlos de Abreu

**Affiliations:** 1 Laboratório de Escrita Científica, Departamento de Morfologia e Fisiologia, Faculdade de Medicina do ABC, Santo André, SP, Brasil; 2 Departamento de Clínica Médica, Disciplina de Cardiologia, Faculdade de Medicina do ABC, Santo André, SP, Brasil; 3 Disciplina de Cirurgia Vascular, Faculdade de Medicina do ABC, Santo André, SP, Brasil; 4 Division of Endocrinology, Metabolism and Diabetes, University of Utah, School of Medicine, Salt Lake City, Utah, USA; 5 Escola de Artes, Ciência e Humanidades da Universidade de São Paulo (USP), São Paulo, SP, Brasil

## Correction

Following the publication of this article [1], we observed that a co-author, Dr. Weslei de Souza, was absent from the authors list. The submitting authors would like to apologise to Dr. Weslei de Souza for this mistake. We have updated the Authors' contributions and Competing interests sections in order to reflect this correction.

## Competing interests

The authors declare that they have no competing interests.

## Authors' contributions

MF, VEV, JRC, CF, AM, CFF, JRB, JAC, CBMM., HMJ, NM, WS and LCA designed research, analyzed data, and wrote the paper. All authors read and approved the final manuscript.
